# Tumor-Specific Classification of Canine Cutaneous and Subcutaneous Tumors Using Heat-Diffusion Imaging and Artificial Intelligence

**DOI:** 10.3390/vetsci13090932

**Published:** 2026-09-09

**Authors:** Gillian Dank, Tali Buber, Liron Levy-Hirsh, Michael S. Kent, Uri Greenberg, Yael Harpaz, Erez Hanael

**Affiliations:** 1 HT Vet Ltd., Hod Hasharon 4532532, Israel; 2 HT Vet Ltd., London NW6 3QT, UK; 3 Department of Surgical and Radiological Sciences, School of Veterinary Medicine, University of California Davis, Davis, CA 95616, USA

**Keywords:** canine cutaneous tumors, dynamic thermography, Heat-Diffusion Imaging, artificial intelligence, mast cell tumor, lipoma, tumor classification

## Abstract

Introduction: Cutaneous and subcutaneous tumors are frequently encountered in canine patients, posing diagnostic challenges in general veterinary practice. Heat-Diffusion Imaging (HDI) is an active dynamic thermography technique that evaluates tissue thermal changes following controlled stimulation. Integration of HDI with artificial intelligence (AI) provides a non-invasive approach for tumor classification. Objective: Our objective was to develop and internally validate HDI-based, tumor-specific AI classifiers for differentiating canine mast cell tumors (MCTs) and lipomas from other cutaneous and subcutaneous masses. Methods: This study included 669 dogs with 1010 cutaneous and subcutaneous masses. Dynamic thermal data were recorded from each mass using an HDI system. This was followed by cytological or histopathological diagnosis. Thermal features were extracted from the recordings and used to develop, train, and internally validate a primary AI-based classifier that distinguishes benign from malignant lesions. A diagnostically confirmed subgroup was used to train and internally validate tumor-specific classifiers for lipomas and mast cell tumors. The diagnostic performance of all classifiers was independently evaluated. Results: The primary classifier cohort comprised 952 masses. Of these, 760 masses were assigned to the training cohort and 192 masses to the test cohort. The primary classifier achieved a sensitivity of 92% (95% CI 83.6–96.3) in the test set of 192 masses, and a prevalence-adjusted negative predictive value (NPV) of 98.1% at an assumed malignancy prevalence of 15%. Among the 55 masses assessed by the lipoma classifier, sensitivity was 86.4% (95% CI 73.3–93.6) and specificity 100% (95% CI 74.1–100); all 38 masses flagged as lipoma were lipomas. Among the 81 masses assessed by the MCT classifier, sensitivity was 31.8% (95% CI 20.0–46.6) and specificity 94.6% (95% CI 82.3–98.5); all 16 masses flagged as MCT were malignant, and 14/16 were diagnosed as MCTs. Conclusions: These findings demonstrate that combining HDI-derived thermal features with AI enables promising, non-invasive differentiation of common canine cutaneous tumor types. The two-tiered diagnostic approach improves upon the primary classifier by providing tumor-specific classification. This strategy may be particularly valuable in general practice settings, where access to immediate cytological or histopathological evaluation can be limited.

## Simple Summary

Skin and subcutaneous lumps are common in dogs but often require invasive sampling to distinguish benign from malignant disease. We tested a non-invasive device, Heat-Diffusion Imaging (HDI), which combines controlled thermal stimulation with artificial intelligence to classify masses without a needle or biopsy. Using data from 1010 masses in 669 dogs, we developed a primary classifier to distinguish benign from malignant lesions, followed by two tumor-specific classifiers targeting lipomas and mast cell tumors, the most common benign and malignant skin tumors in dogs, respectively. The primary classifier reliably ruled out malignancy, correctly identifying malignant masses with a sensitivity of 92% and a negative predictive value of 98%. The lipoma classifier confirmed benign lipomas with high confidence and no false positives, while the mast cell tumor classifier flagged a smaller subset of lesions but did so with high specificity, with nearly all flagged masses confirmed malignant and most confirmed as mast cell tumors, helping prioritize cases needing urgent follow-up. These findings suggest that combining dynamic thermal imaging with AI offers a practical triage tool to guide clinical decision-making, though cytology or histopathology remains necessary for definitive diagnosis.

## 1. Introduction

Cutaneous and subcutaneous tumors represent one of the most common categories of neoplasms in dogs and are frequently encountered in both general and referral veterinary practices [1,2]. Although histopathology remains the diagnostic gold standard, its invasive nature, associated costs, and processing delays limit routine use. Fine-needle aspiration is more commonly performed due to its simplicity and low risk; however, diagnostic yield may be insufficient or inconclusive, particularly when sample quality is compromised. This highlights the need for an alternative, non-invasive, simple-to-use procedure for triage of cutaneous and subcutaneous masses [3,4].

Medical thermography is a non-invasive imaging technique that uses infrared detection to capture and visualize temperature patterns on the surface of living tissues, providing insight into physiological abnormalities such as inflammation, disrupted circulation, nerve impairment, or disease-related changes [5,6,7,8,9]. In oncology, its use is based on the observation that neoplastic tissues often demonstrate physiological changes, including angiogenesis, vasodilation, nitric oxide-mediated vascular signaling, increased capillary density, and heightened metabolic activity, which may alter their thermal properties compared to healthy tissues. In addition to specific changes in the tumor, including necrosis, reduced vascularization, or encapsulation, the thermal measurements are further affected by external and anatomical factors such as ambient temperature, hair coat, clipping, lesion depth, inflammation, regional anatomy, and could also be affected by operator variability. Thermography is generally classified into two approaches: traditional (passive) thermography, which records naturally emitted, steady-state surface temperatures using infrared imaging, and active (dynamic) thermography, which involves the application of a controlled thermal stimulus, such as brief heating or cooling, followed by assessment of tissue responses during the recovery phase [5,9,10,11].

In veterinary medicine, thermographic research has largely relied on passive infrared imaging to assess steady-state surface temperatures, most often comparing benign and malignant lesions. Studies evaluating canine tumors using passive thermography have reported variable findings across tumor types, with lesions described as either hyperthermic or hypothermic. This inconsistency may partly reflect a limitation of the passive approach, because it captures only a single static snapshot of baseline surface temperature; it may be less able to detect the functional, time-dependent thermoregulatory responses that could help differentiate neoplastic from non-neoplastic tissue. While these observations support the premise that neoplastic processes are associated with altered thermal properties, their inconsistency underscores the limitations of passive thermography as a standalone diagnostic approach [12,13,14,15].

To address these limitations, active (dynamic) thermography has been proposed as an alternative methodology. By applying a controlled thermal stimulus and analyzing tissue rewarming or cooling kinetics, dynamic thermography interrogates deeper physiological processes, including tissue perfusion, vascular responsiveness, and metabolic activity, potentially enhancing diagnostic sensitivity and reproducibility. This approach has gained traction in human oncology, particularly in the assessment of breast and soft tissue tumors, where dynamic thermal responses revealed underlying metabolic and vascular differences [16,17].

The increasing integration of advanced imaging modalities and artificial intelligence (AI) has created new opportunities for non-invasive tumor assessment. Machine learning may be well suited to analyzing the multidimensional thermal patterns generated by dynamic thermography, as it can identify non-linear relationships among multiple thermal features simultaneously, rather than relying on a single predefined cutoff. This complex model can enhance the ability to differentiate between benign and malignant tumors, based on their unique thermal characteristics [18,19].

Heat-Diffusion Imaging (HDI) is a thermography-based technique that incorporates both passive (steady-state) and active dynamic imaging to evaluate thermal recovery patterns associated with tumor biology in dermal and subcutaneous canine masses. The HDI system uses a standardized protocol involving controlled heating followed by spontaneous cooling to acquire steady-state and dynamic thermal data, thereby minimizing environmental variability. Previous veterinary studies have demonstrated that HDI can capture relevant thermal signatures and differentiate benign from malignant lesions, yielding a sensitivity, specificity, and negative predictive value (NPV) of 85–93%, 67–88%, and 95–97%, respectively [18,19].

This study describes the development and internal validation of HDI-based machine-learning tumor-specific classifiers to distinguish lipomas and mast cell tumors from other common canine skin and subcutaneous tumors.

## 2. Materials and Methods

### 2.1. Study Population

This training and validation study used prospective data collected from client-owned dogs presenting with cutaneous or subcutaneous masses at 13 participating clinical sites across Israel, Italy, Switzerland, Slovenia, the United States, and Germany between April 2022 and February 2026. The Sites that participated in the multicenter data-collection program included six private general practices, five private referral centers, and two university teaching hospitals. All owners provided informed consent, and the National Health Ministry ethical review board approved the study (Approval Number: PET-2023-03-NE and HU-NER-2020-015-A).

Inclusion criteria required that masses be externally visible or palpable, with sufficient surrounding healthy tissue available for comparative imaging, and that the dog be a suitable candidate for fine needle aspiration of the mass. Exclusion criteria were mammary, facial, or testicular tumors for practical reasons and masses whose overlying skin was ulcerated, bleeding, scabbed, severely inflamed, or otherwise compromised, since loss of skin integrity compromises the reliability of thermal data in these contexts. Masses with intact overlying skin were therefore enrolled irrespective of the diagnosis subsequently obtained, including 58 masses later diagnosed as inflammatory processes. Since an inflammatory process is not a neoplasm, these masses were not assigned to the benign or malignant analysis cohorts and were instead evaluated separately as a challenge group. To maintain data integrity, only the first complete scan of each mass was included in the analysis. Masses whose scan was interrupted by movement or technical fault were excluded without rescanning. Interruption occurred before any diagnostic information was available, so exclusion was independent of the final diagnosis.

### 2.2. The HDI System

The HDI system (model HT Vista; HT Vet Ltd., Hod Hasharon, Israel consists of a control unit with an integrated compute module, a touch-screen interface, proprietary software (version 1.14), and a handheld probe [18,19]. This probe includes an optical camera, two high-power LED emitters (10 W each) operating at 460 nm, which serve as the heating source, and a long-wave infrared (LWIR) thermal camera (FLIR Lepton 3.5; Teledyne FLIR LLC, Wilsonville, OR, USA) with a resolution of 160 × 120 pixels (19,200 pixels) and sensitivity below 50 mK. The field of view is 6.5 × 4 cm. An internal thermal control maintains the focal plane array at 35 °C to minimize environmental influence and improve thermal image quality. Before each scan, the system performs a flat field correction to normalize pixel sensitivity and optimize thermal capture. Each unit is additionally validated during manufacture against a reference phantom that verifies delta heating and characterizes thermal camera and LED noise, with 20 repeated scans acquired per device to confirm signal reproducibility before release.

The device is CE-marked under Regulation (EU) 2017/745 and has undergone electrical safety testing to IEC 60601-1, electromagnetic compatibility testing to IEC 60601-1-2, light source safety testing to IEC 60601-2-57, photobiological safety assessment to IEC 62471, and usability evaluation to IEC 60601-1-6 with IEC 62366-1. Scanning is non-invasive and involves no ionizing radiation.

### 2.3. The Scanning Process and Data Collection

All dogs were manually restrained, and the fur over the mass and adjacent healthy tissue was clipped to ensure proper tissue heating and imaging. The clipped area was then scanned by the HDI system. Each scan consists of three consecutive phases lasting a total of 42 s: a steady-state phase of 2 s (basal temperature is recorded), an active heating phase of 10 s (a controlled LED stimulus gently warms the tissue), and a passive cooling phase of 30 s (thermal recovery is recorded to capture temperature decay characteristics).

The handheld probe maintains direct contact with the skin throughout the scan, creating a sealed microenvironment that minimizes the effects of airflow, ambient temperature, and external heat sources. HDI is an active dynamic technique, and all extracted parameters are differential, describing the thermal response of the mass relative to adjacent healthy tissue within the same acquisition rather than absolute surface temperature. The measurement is therefore not referenced to ambient conditions. Room temperature, humidity, and a standardized acclimatization period were therefore not recorded or controlled. Hair overlying the mass and the adjacent reference area was clipped immediately before scanning, typically within one to two minutes, although this interval was not recorded for individual scans. No other lesion preparation was applied. Scans were performed in both conscious and sedated or anaesthetized animals. Sedation status was not recorded for individual scans. Systemic changes in perfusion are expected to shift the mass and the adjacent reference tissue in the same direction and to a similar degree, leaving the difference between them, on which all parameters are based, largely unchanged.

**Figure 1:**
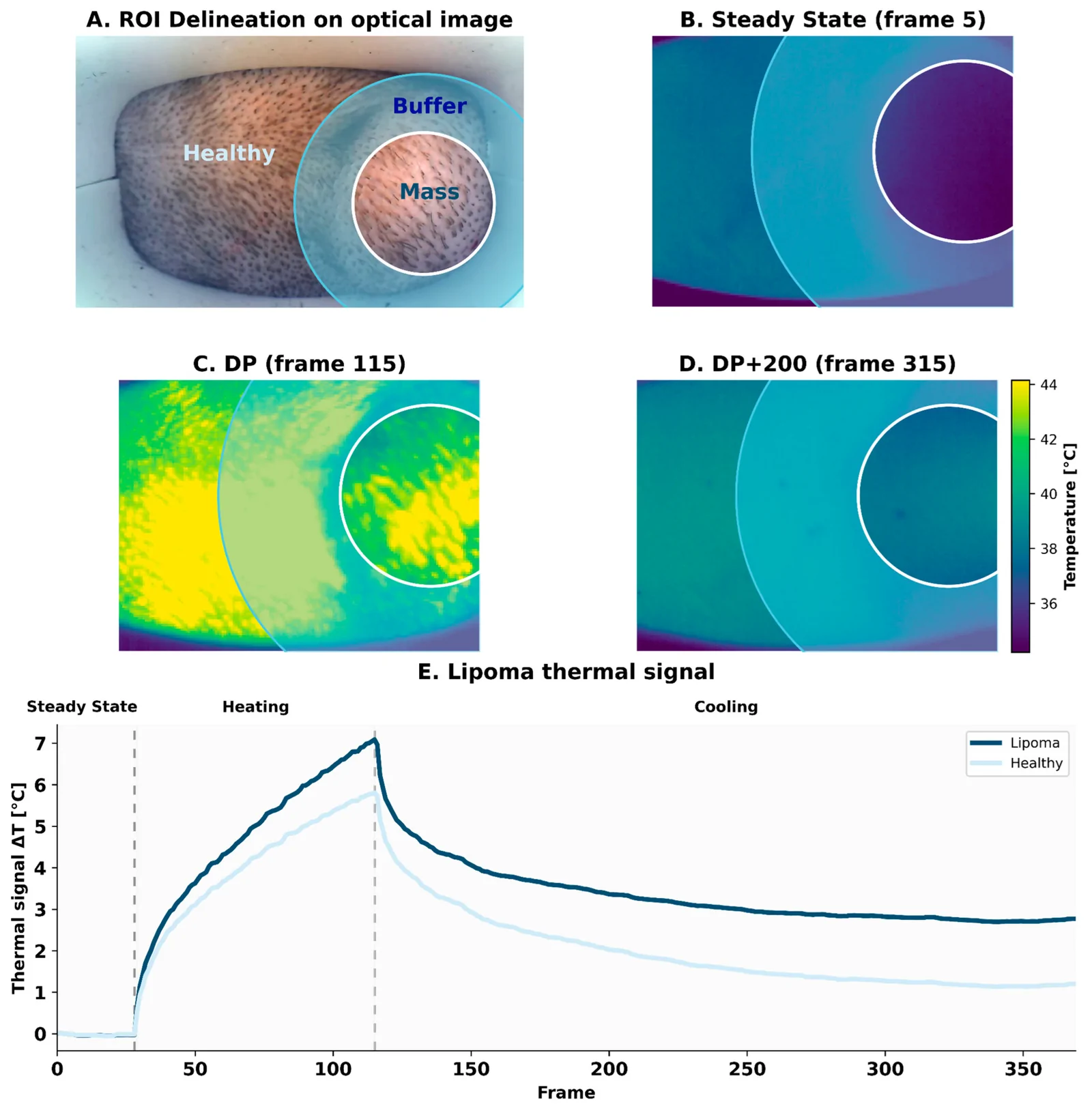
Optical and thermal analysis of a lipoma region of interest (ROI). (**A**) Optical image with mass (white circle), buffer region (blue shaded annulus), and healthy tissue delineation. (**B**–**D**) Representative thermal frames at steady state (frame 5), end of heating/decay point (DP) (frame 115), and cooling (frame 315) with corresponding marking of the regions as detailed in the optical image. (**E**) Mean thermal response (ΔT) of lipoma and healthy tissue over time, highlighting distinct heating and cooling dynamics. The temperatures are normalized to the steady-state temperature to better view the difference between the ROIs.

Once the scan is completed, an optical image of the scanned area appears on the touch screen interface. The clinician manually identifies and delineates three regions of interest (ROIs): (i) the tumor region (an inner circle), (ii) the buffer region (a peritumoral margin), and (iii) the healthy skin region (an outer reference area) (Figure 1). In cases where the thermal image shows a clearer boundary than the optical view, these annotated ROIs are then refined algorithmically before feature extraction. The refined ROIs serve as the input for thermal feature extraction and classification. Thermal data are securely transmitted to a cloud platform and analyzed using a machine-learning algorithm that generates near-real-time results.

After the scan, fine-needle aspiration samples were collected and submitted to an external commercial reference laboratory, where each sample was read by a single clinical pathologist, blinded to the scan results; no formal multi-reader consensus review was applied. Where available, histopathology was used to confirm the diagnosis. Tumor grade was assigned by the reporting laboratory using standard grading systems and recorded when available. Diagnostic adjudication followed a consistent hierarchy. Where both cytology and histopathology were available, and the diagnoses were discordant, the histopathological diagnosis was taken as definitive. A non-diagnostic cytological result was never carried forward as a diagnosis: such masses were excluded unless histopathology subsequently provided a definitive diagnosis, in which case the histopathological result served as the reference standard. Masses without a definitive diagnosis by either method were excluded from all analyses.

**Figure 2:**
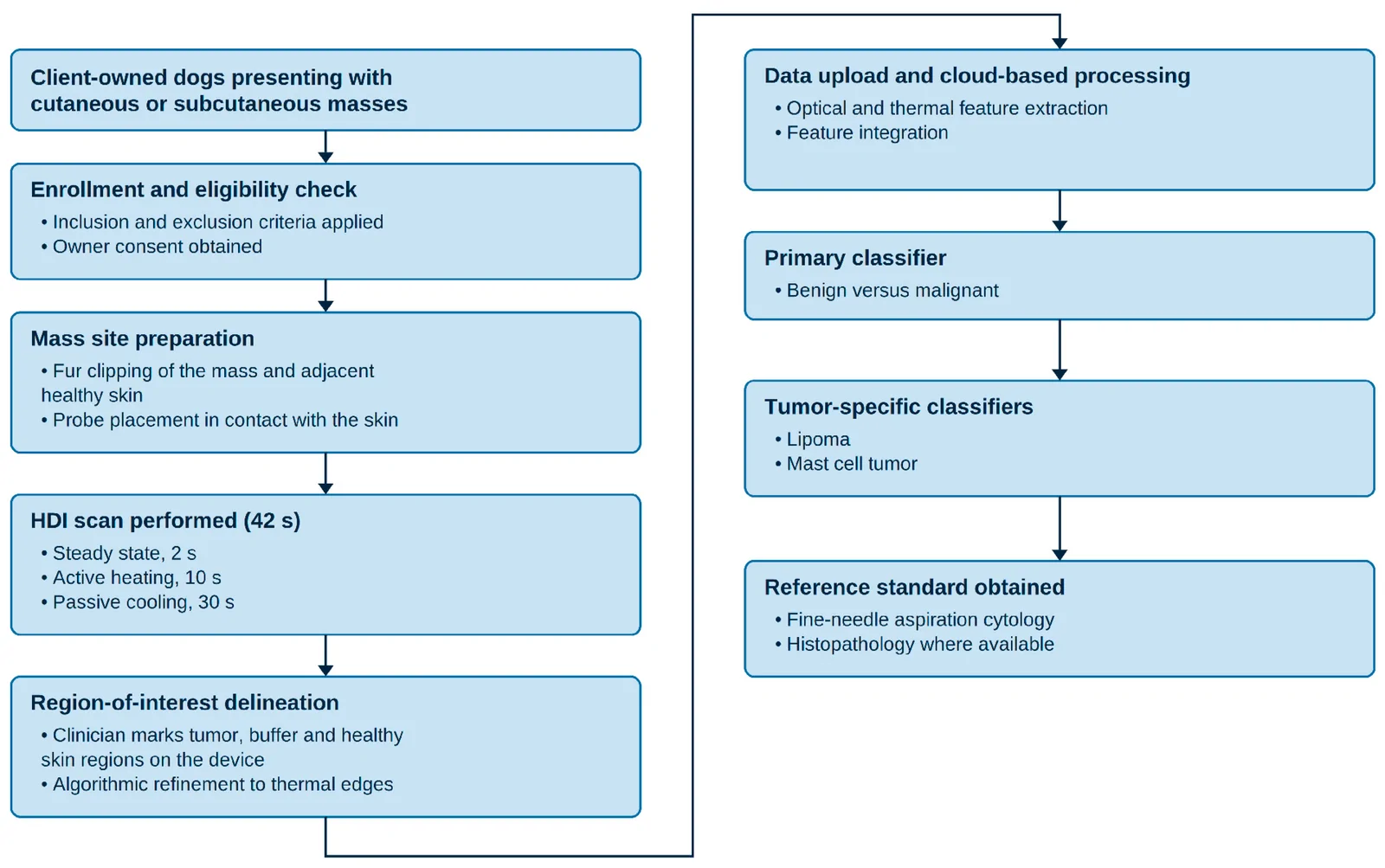
Study workflow, from enrollment to reference diagnosis. HDI, heat diffusion imaging.

To ensure unbiased data collection, the HDI system’s classifications did not influence diagnostic or treatment decisions. All patient data and diagnostic results were recorded and managed using a MongoDB Atlas cloud database (version 8.0; MongoDB Inc., New York, NY, USA) (Figure 2).

### 2.4. Adverse Events

Safety was assessed at the time of scanning and for 24 h thereafter. An adverse event was defined as any local or systemic reaction attributable to the scan, including erythema, local irritation, edema, pain on palpation of the scanned site, thermal injury, or signs of distress during or immediately after the procedure. The scanned site was inspected by the attending clinician immediately after each scan. Owners were advised to monitor the dog for the following 24 h and to contact the practice if any abnormality of the scanned site or any change in the dog’s behavior was observed.

### 2.5. Primary and Tumor-Specific Classifier’s Workflow

The HDI system relies on several unique algorithms built in a structured pipeline: (1) pre-processing, (2) feature extraction, (3) primary classification (training/validation and test), and (4) tumor-specific classification (training/validation and test).

### 2.6. Pre-Processing—Real-Time Scan Optimization

Real-time algorithms, including fur detection, motion detection, heated area validation, and skin segmentation, operate simultaneously during the scan, ensuring only high-quality data are captured by filtering artifacts and optimizing thermal signal extraction. The fur detection algorithm identifies residual fur at the onset of the scan, preventing data acquisition if the area is insufficiently clipped. The movement detection algorithm automatically rejects scans containing excessive movement. The heated area validation algorithm verifies adequate tissue heating during acquisition. The scan is aborted if the data collected is insufficient for analysis. The skin segmentation algorithm distinguishes skin from fur or other background interference, constraining the analysis to relevant tissue within the scan area.

### 2.7. Feature Extraction and Representation

For mass classification, features were explicitly constructed from both thermal and optical data acquired from three manually defined regions of interest (ROIs): tumor, peritumoral buffer, and adjacent healthy skin. Feature extraction was performed separately for each of the three thermal acquisition phases: steady-state, heating, and cooling.

From the thermal data, quantitative features were computed directly from temperature maps at specific time points and over defined temporal intervals. These included first-order statistical descriptors (mean, median, standard deviation, and interquartile range) of temperature within each ROI, as well as spatial descriptors such as temperature gradients and intra-ROI variance. During the heating phase, temporal features describing heat absorption dynamics were calculated, including temperature rise rate and spatial diffusion patterns. During the cooling phase, thermal decay characteristics were quantified using recovery rate, temperature retention, and decay slope metrics derived from time–temperature curves.

To capture spatial–temporal thermal patterns not adequately described by the handcrafted descriptors, a convolutional encoder–decoder network (U-Net; three resolution levels) was trained to delineate small focal thermal features within individual thermal maps. Training targets were derived from point annotations of thermal maps sampled from the decay phase; each annotated point was automatically expanded into a small pixel-level region. Training minimized a per-pixel binary cross-entropy loss computed only over annotated pixels, with an additional term penalizing excessive predicted area to discourage over-segmentation. Data were partitioned at the scan level, so that frames from the same scan never appeared in both partitions; training hyperparameters are given in Supplementary Table S1. The network was applied independently to each map of the decay sequence, and its outputs were thresholded to binary detections. Detections corresponding to the same structure were then linked across the sequence, allowing the temporal behavior of each structure to be quantified. The resulting scalar descriptors served as classifier inputs rather than as a standalone prediction. No probability calibration was applied at this stage since the descriptors derive from binary detections rather than probabilities.

To incorporate optical surface appearance alongside the handcrafted thermal descriptors, a self-supervised Vision Transformer backbone (DINOv2, ViT-S/14) was fine-tuned to classify each mass as cutaneous or subcutaneous from its visible-light image. Each scan contributed two inputs, a segmentation-centered 200 × 200 mass patch and the full 760 × 460 optical frame, each resized to 224 × 224 and passed through a single shared backbone; the two resulting feature vectors were concatenated and mapped by a linear head to the cutaneous-versus-subcutaneous output, so that both local lesion morphology and its surrounding context contributed to the prediction. Training hyperparameters are given in Supplementary Table S2. In deployment, the model output is used as an additional input feature, combined with the handcrafted thermal descriptors for the downstream classifier.

All extracted features, thermal statistical measures, temporal decay descriptors, mass size parameters, and the descriptors derived from the focal thermal detection and optical depth networks were aggregated into a unified feature vector. Feature values were normalized across patients and anatomical regions before being passed to the downstream classification algorithm.

### 2.8. Primary Classification

**Figure 3:**
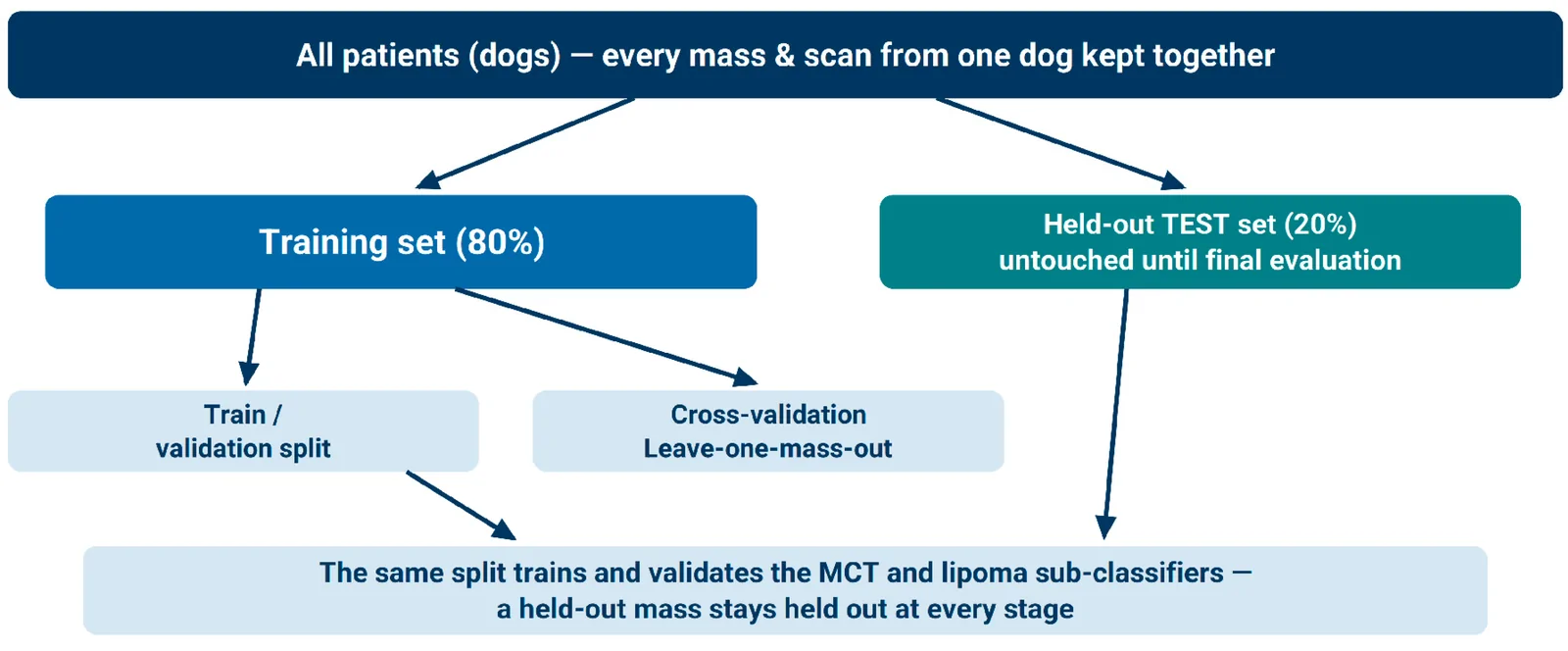
Dog-level split: every mass and scan from one dog stays in a single set; the held-out test set is untouched until final evaluation, and the same split is reused for the mast cell tumor and lipoma sub-classifiers, so a held-out mass stays held out at every stage. Model, features, and thresholds were fixed on the training data only, and the held-out test set provides the unbiased final estimate.

The primary classification model was designed to differentiate benign from malignant tumors, with a target sensitivity of 90 percent to minimize false negatives. The 952 masses with a definitive neoplastic or non-neoplastic diagnosis, contributed by 626 dogs, were divided into training and test sets at an 80:20 ratio of masses, subject to the constraint that all masses from a given dog were assigned to the same set, so that no dog contributed masses to both. Assignment was stratified by tumor class and by cutaneous versus subcutaneous location. This yielded 760 training masses from 501 dogs (760/952, 79.8%) and 192 held-out masses from 125 dogs (192/952, 20.2%). Lesions diagnosed as inflammatory processes (*n* = 58), owing to their variable thermal signatures, were excluded from this split and evaluated separately as a challenge group, giving 1010 masses from a total of 669 dogs. The training portion was further divided into training and validation subsets during model development, and the held-out test set was not examined until final evaluation. The model output was a malignancy probability score. Classification thresholds were fixed in advance: they were selected on the training data alone as the operating point achieving the target sensitivity of 90% with the highest corresponding specificity, and were locked before the held-out test set was examined. They were then applied unchanged to the held-out set and were therefore not optimized for test-set performance. Masses scoring above the threshold were classified as malignant (Figure 3).

### 2.9. Tumor-Specific Classification

The tumor-specific classifier aimed to identify lipomas and MCTs among masses initially classified as likely benign or malignant, respectively. The goal for both tumor-specific classifiers was to achieve a minimum specificity of 90 percent. Only cases with high or low malignancy probability scores were included in this analysis. The filtered dataset was then divided into training and test sets at an 80:20 ratio. The same test set used for the primary classifier was used to evaluate tumor-specific classifier performance, allowing consistent comparison across models.

### 2.10. Statistical Analysis

Descriptive statistics were calculated for patient demographics, tumor location, and tumor size. Gaussian mixture models were used to examine the distribution of thermal features and identify patterns associated with specific tumor types. Cross-validation and leave-one-mass-out testing were used to assess the reliability and generalizability of the model across the dataset. Performance metrics included sensitivity, specificity, accuracy, positive predictive value (PPV), and NPV, calculated separately for both the primary and tumor-specific classifiers as follows:

Diagnostic accuracy

Sn = TP/(TP + FN)(1)

Sp = TN/(TN + FP)(2)

PPV = TP/(TP + FP)(3)

NPV = TN/(TN + FN)(4)

Accuracy = (TP + TN)/(TP + TN + FP + FN)(5)

Wilson score confidence interval

Ninety-five percent confidence intervals for all proportions were calculated by the Wilson score method, which remains valid for proportions close to 0 or 1 and for small samples [20]. For *k* events in *n* observations, with $\hat{p}$ = *k*/*n* and *z* = 1.96:

(6) $$\mathrm{CI}=[\hat{p}+z^{2}/(2n)\pm z\surd \left(\hat{p}(1-\hat{p})/n+z^{2}/\left(4n^{2}\right)\right)]/\left(1+z^{2}/n\right)$$

Likelihood ratios

LR+ = Sn/(1 − Sp)(7)

LR− = (1 − Sn)/Sp(8)

The predictive values of diagnostic tests are contingent upon disease prevalence within the population. The malignancy prevalence in this study population (29.5%, 298/1010) is higher than that expected in first-opinion practice; Therefore, predictive values calculated directly from the observed counts would not transfer to the intended-use setting. Predictive values were therefore standardized by applying the observed sensitivity and specificity to an assumed malignancy prevalence *p*:

PPV(*p*) = (Sn × *p*)/[Sn × *p* + (1 − Sp) × (1 − *p*)] (9)

NPV(*p*) = [Sp × (1 − *p*)]/[Sp × (1 − *p*) + (1 − Sn) × *p*] (10)

In this study, we assumed a malignancy prevalence of 15% (*p* = 0.15), a conservative upward rounding of the 10.8% prevalence observed across the general-practice sites in this study. Literature-derived prevalence estimates were not used because they derive from masses submitted for biopsy and therefore select for malignancy [1,21]. Across assumed prevalence of 10.8% to 20%, the standardized negative predictive value of the primary classifier ranged from 98.7% to 97.3% and the positive predictive value from 28.3% to 44.9%. Standardized predictive values are reported as point estimates derived from the observed sensitivity and specificity. For consistency, the corresponding values are presented in the Results and Discussion as the prevalence-adjusted PPV and NPV. Predictive values for the tumor-specific classifiers are reported as observed within the masses assessed by each classifier, and the prevalence of the target diagnosis in that group is stated alongside them. These values are therefore specific to that population and are not standardized to an assumed prevalence, as was done for malignancy in the primary classifier. Likelihood ratios, which are independent of prevalence, are reported in Supplementary Table S3 to allow the predictive values to be recalculated for other settings. All analyses were implemented in Python (version 3.11.2) using the NumPy (1.24.2), SciPy (1.15.3), pandas (2.3.0), and scikit-learn (1.7.2) libraries.

Due to the limited size of the test set in the tumor-specific classifiers, results are presented for the combined training and test sets, as well as separately, to provide more robust estimates of classifier performance.

### 2.11. Use of Generative AI

Generative AI (Claude, Anthropic; Opus 4.8) was used to perform statistical calculations from the study dataset and to draft and edit sections of the manuscript text and generate figures and tables. The analyses were specified by the authors, and all reported values were verified against the source dataset by the authors, who take full responsibility for their accuracy. Generative AI was not involved in data collection, in the development or training of the classification algorithms, or in the assignment of reference diagnoses.

## 3. Results

### 3.1. Study Population

The study included 1010 masses from 669 dogs, of which 289 were mixed-breed, and 380 were purebred dogs, with the most common breeds including Labrador Retrievers (51), Shih Tzus (22), Golden Retrievers (21), American Pit Bull Terriers (18), French Bulldogs (15), Boxers (14), and German Shepherds (14). For all other breeds, sample sizes were fewer than 10 individuals. Of the 669 dogs, 353 were females (52.8%), of which 278 (78.8%) were neutered, and 316 were males (47.2%), of which 226 (71.5%) were neutered. The median age was 9 years (range 1–15). The median number of tumors per patient was 1 (range 1–9), with 474 dogs presenting with 1 tumor, 116 dogs with 2 tumors, 42 dogs with 3 tumors, 21 with 4 tumors, 11 with 5 tumors, 3 dogs with 7 tumors, and 2 dogs presenting with 9 tumors each.

Masses were collected almost equally, with 510 (50.5%) from general practice and 500 (49.5%) from referral centers, contributed by 345 and 324 dogs, respectively. Malignancy prevalence differed markedly between settings, with 10.8% (55/510) in general practice and 48.6% (243/500) at referral (chi-square test, *p* < 0.001). Accounting for repeated masses within dogs using logistic regression with cluster-robust standard errors gave an odds ratio of 7.82 (95% CI 5.19 to 11.78, *p* < 0.001).

The 1010 masses were distributed across five anatomical regions: the trunk, back, pelvis and neck (397, 39.3%), the legs (257, 25.4%), the axilla, inguinal region and abdomen (249, 24.7%), the perianal region and tail (64, 6.3%) and the head (43, 4.3%). Subcutaneous masses accounted for 582 of 1010 (57.6%) and cutaneous masses for 428 (42.4%).

Diagnostic confirmation was obtained via cytology in 715 masses and histopathology in 295 masses. In 42/295 (14.2%) masses, both cytology and histopathology were performed. Cytology was non-diagnostic in 9/42 (21.4%) masses. Among the 33 masses (78.6%) for which cytology yielded a diagnosis, cytology and histopathology agreed in 32/33 (97.0%), including complete agreement for mast cell tumors (20/20), benign adnexal/epidermal cysts and tumors (4/4), lipomas (3/3), and carcinoma (1/1), and partial agreement for soft tissue sarcomas (4/5). The single discordant mass was read as hyperplasia on cytology and as a soft tissue sarcoma on histopathology; the histopathological diagnosis was used.

In total, 654 tumors were benign, 298 were malignant, and 58 were inflammatory processes. Lipomas, benign adnexal tumors, and sebaceous tumors accounted for the majority of benign diagnoses, whereas MCTs and soft tissue sarcomas represented the most frequent malignant diagnoses (Table 1).

### 3.2. Primary Classifier Results

The primary classifier study group comprised 952 masses. Of these, 760 masses were assigned to the training set and 192 masses to the test set. When applied to the test set, the primary classifier correctly identified 84/117 benign cases as true negatives and 69/75 malignant cases as true positives, resulting in an overall accuracy of 79.7% (153/192), a sensitivity of 92.0% (95% CI, 83.6–96.3), a specificity of 71.8% (95% CI, 63.0–79.2), and an area under the receiver operating characteristic curve (AUC) of 0.916 (95% CI, 0.867–0.955). Performance on the training set was comparable, with an AUC of 0.884 (95% CI, 0.857–0.908), indicating no evidence of overfitting. The prevalence-adjusted NPV was 98.1%, and the prevalence-adjusted PPV was 36.5%. Inflammatory lesions were flagged as malignant in 42 out of 58 cases (72.4%). The distribution of diagnoses and the proportion of masses correctly classified within each tumor class are presented for benign diagnoses in Table 2 and for malignant diagnoses in Table 3, separately for the training and test sets. Full confusion matrices and the corresponding performance metrics for all three classifiers, in the training, test, and combined sets, including likelihood ratios, are provided in Supplementary Table S3.

### 3.3. Tumor-Specific Classifier

For the training and validation of the tumor-specific classifier, 636/1010 masses were selected based on their primary classifier probability scores. Only cases with probability scores above a predefined threshold for either benign or malignant classification were included.

#### 3.3.1. Lipoma-Classifier Results

The lipoma classifier was trained on 251 masses that had been classified as benign by the primary classifier, including 214 lipomas. In the test set of 55 masses classified as benign by the primary classifier, of which 44 were lipomas, the classifier achieved a sensitivity of 86.4% (38/44, 95% CI 73.3–93.6), a specificity of 100% (11/11, 95% CI 74.1–100.0), and a PPV of 100% (38/38, 95% CI 90.8–100.0) for the identification of lipomas. When the training and test sets were combined, the classifier correctly identified 220 of 258 lipomas, achieving an overall sensitivity of 85.3% (220/258, 95% CI 80.4–89.1), a specificity of 93.8% (45/48, 95% CI 83.2–97.9), and a PPV of 98.7% (220/223, 95% CI 96.1–99.5) (Supplementary Table S3).

In the test set, all 38 masses flagged as lipoma were benign (100%, 95% CI 90.8–100.0). No malignant mass was flagged as a lipoma in the test set. When the training and test sets were combined, 220/221 flagged masses were benign, giving a negative predictive value for malignancy of 99.5% (95% CI 97.5–99.9); the single exception was a soft tissue sarcoma. Standardized to an assumed malignancy prevalence of 15%, the negative predictive value of a lipoma flag was 99.8%, compared with 98.0% for a benign classification by the primary classifier alone, corresponding to a reduction in malignancy risk from 2.0% to 0.2%. This comparison rests on a single malignant mass, and its confidence interval overlaps that of the primary classifier.

#### 3.3.2. MCT-Classifier Results

The MCT classifier was trained on 249 samples, already classified by the primary classifier as malignant based on their probability scores, including 101 MCTs. In the test set of 81 masses assessed by the MCT classifier, of which 44 were MCT, the classifier achieved a sensitivity of 31.8% (14/44, 95% CI 20.0–46.6), a specificity of 94.6% (35/37, 95% CI 82.3–98.5), and a PPV of 87.5% (14/16, 95% CI 64.0–96.5) for the identification of mast cell tumors. When the training and test sets were combined, the classifier correctly identified 55 of 145 mast cell tumors, achieving a sensitivity of 37.9% (95% CI 30.4–46.0), a specificity of 91.4% (169/185, 95% CI 86.4–94.6), and a PPV of 77.5% (55/71, 95% CI 66.5–85.6) for a MCT diagnosis. Therefore, a negative MCT-specific result does not rule out mast cell tumor (Supplementary Table S3).

In the test set, all 16 masses flagged as MCT were malignant (100%, 95% CI 80.6–100.0), comprising 14 mast cell tumors and two soft tissue sarcomas. When the training and test sets were combined, 467 of 952 masses (49.1%) were classified as malignant by the primary classifier. Of these, 71 received an MCT flag, and 396 did not. Malignancy was confirmed in 62/71 flagged masses (87.3%, 95% CI 77.6–93.2) and in 212/396 masses classified as malignant without a flag (53.5%, 95% CI 48.6–58.4). This corresponds to an odds ratio for malignancy of 5.98 (95% CI 2.89–12.36). Both proportions reflect the malignancy prevalence of the study group (298/952, 31.3%) and were therefore standardized to an assumed prevalence of 15% malignant masses. Under that assumption, a malignant classification by the primary classifier without an MCT flag corresponds to a 30.9% probability of malignancy, and a malignant classification with an MCT flag corresponds to 72.7%.

### 3.4. Adverse Events

No adverse events were observed at the time of scanning, and none were reported subsequently.

## 4. Discussion

In this study, we present an updated HDI system that includes an upgraded primary benign–malignant classifier for canine cutaneous and subcutaneous tumors and newly developed tumor-specific classifiers for lipomas and MCTs.

The primary classifier is designed to screen for malignancy in the general practice setting, where most of the cutaneous and subcutaneous tumors are benign [1]. The reported benign-to-malignant ratio in canine cutaneous and subcutaneous tumors varies between studies, reaching as high as 70:30, with a consistent reported predominance of benign tumors over malignant ones [1,21]. However, as most studies rely on data collected from diagnostic laboratories and given that benign lesions are frequently asymptomatic, undocumented, and diagnosed in-house, the actual prevalence of benign tumors in the general population is likely much higher than reported [21]. Consistent with this, our results from general practice showed that only 10.8% of evaluated masses were malignant, further highlighting the predominance of benign tumors in these settings. Therefore, our analysis used a benign-to-malignant ratio of 85:15 to compensate for the reporting bias, and our predictive metrics (i.e., NPV and PPV) were adjusted to fit this proposed ratio.

A useful screening tool in a low-prevalence population should be highly sensitive and have a high NPV, allowing the clinician to safely rule out a disease. In this study, the primary classifier achieved a sensitivity of 92%, a significant improvement over the 85% sensitivity previously reported [18,19]. While its specificity and prevalence-adjusted PPV were more modest (71.8% and 36.5%, respectively), an expected finding in low-prevalence screening contexts, its prevalence-adjusted NPV was 98.1%. In the general practice setting, which has a low prevalence of malignancy, this high NPV can provide confidence in ruling out malignancy and guiding clinical decision-making.

The tumor-specific classifiers were designed to identify lipomas and MCTs within a subgroup of tumors already classified by the primary classifier as likely highly benign or highly malignant, respectively. Given that these classifiers follow the sensitive screening done by the primary classifier, specificity and PPV were prioritized over sensitivity to rule in the diagnosis of lipoma or MCT with high certainty.

Accordingly, in the test set (55 masses), the lipoma classifier detected lipomas with high specificity and moderate sensitivity (100% and 86.4%, respectively), with all 38 masses flagged as lipomas confirmed as lipomas. In the training set, the one malignant mass flagged as a lipoma was a low-grade soft tissue sarcoma. This mass was misclassified by the primary classifier as benign, possibly because it was located deep within the axillary tissues and surrounded by subcutaneous fat, which may have attenuated the thermal signal originating from the tumor itself. This observation highlights the influence of tumor depth and surrounding tissue composition on thermal signal interpretation. The predictive value of a lipoma flag is best estimated from the combined training and test sets, as no malignant mass was flagged as a lipoma in the test set alone. Standardized to an assumed malignancy prevalence of 15%, the probability that a mass flagged as a lipoma is benign was 99.8%, compared with 98.0% for a benign classification by the primary classifier alone, reducing the residual probability of malignancy from 2.0% to 0.2%. Together, these predictive values may support the clinician in triaging a scanned mass as likely benign and suggestive of a lipoma, which can help guide case management. However, HDI should not be considered a replacement for cytology or histopathology when these are clinically indicated, as definitive diagnosis still relies on tissue-based confirmation.

In the MCT classifier test set (81 masses), a high specificity of 94.6% and a sensitivity of 31.8% were achieved. This sensitivity is a result of an intentional design trade-off that prioritized specificity and PPV. While appropriate given its role downstream of an already sensitive primary screen, this trade-off limits its utility for comprehensive detection of MCTs: a negative result from the MCT classifier should not be interpreted as ruling out that diagnosis, since the mass remains flagged by the primary classifier for cytological or histopathological follow-up.

The 2 cases misclassified as MCTs were soft tissue sarcomas, suggesting that the classifier preferentially highlights malignant behavior. As no benign mass was flagged in the test set, this effect is best quantified in the combined training and test sets, where an MCT flag raised the standardized probability of malignancy from 30.9% to 72.7% over a malignant classification by the primary classifier alone (odds ratio 5.98, 95% CI 2.89–12.36). This predominance of high-risk lesions highlights the clinical relevance of the MCT classifier in guiding prioritization of cases that require timely diagnostic and therapeutic intervention, while reducing the impact of overclassification. Given the high specificity and relatively low sensitivity observed for this classifier, an elevated MCT probability score may be best interpreted as a high-specificity ‘rule-in’ alert, prompting further cytological evaluation, rather than as a tool for excluding MCT. In addition, an HDI-based diagnosis of MCT provides the dual benefit of enabling antihistamine administration before FNA, possible staging, and encouraging pet owners to consent to further diagnostic evaluation of these lesions.

The thermal signature underlying the MCT classifier likely reflects unique biological features of these tumors. Mast cell tumors are characterized by the release of vasoactive mediators, particularly histamine, which increase local blood flow and vascular permeability [22,23]. In combination with mast cell-driven angiogenesis and peritumoral inflammation, these processes can elevate surface temperature and produce a broader thermal halo. The HDI system appears to capture these integrated physiological responses.

Inflammatory lesions presented a particular diagnostic challenge for the HDI system, with 72.4% being misclassified as malignant by the primary classifier. The most probable explanation for the misclassification is the heterogeneity of inflammatory processes and their overlap with malignant thermal signatures. This tendency toward overclassification is clinically preferable to underdiagnosis. To preserve classifier robustness, inflammatory lesions were excluded from model training and analyzed separately, acknowledging their complex and variable thermal behavior; additional studies are needed to improve their thermal characterization.

The improved performance of the HDI system reflects several key technological advancements. These include new real-time algorithms that enhance thermal data quality, an improved method for marking regions of interest (ROIs), and a refined classification algorithm.

Of these advancements, the improved method for marking ROIs represents a major methodological innovation. The clinician-led spatial annotation of the entire mass enabled the extraction of region-specific thermal features across all scan phases, including steady-state, heating, and cooling. This ROI-based approach represents a significant improvement over earlier systems that relied on small reference points [18,19]. The expanded spatial resolution is likely to have contributed to the refinement of the classification algorithm by allowing the analysis of larger tissue areas, improving feature robustness, and reducing susceptibility to confounding factors such as surface skin conditions and environmental variability.

Another enhancement to the algorithm was the addition of two deep learning components alongside the handcrafted thermal descriptors. A convolutional encoder–decoder network was used to delineate small focal thermal features and quantify their behavior across the decay sequence, and a self-supervised Vision Transformer was used to assess lesion depth from the visible-light image. Both serve as additional classifier inputs rather than as standalone predictions.

Notably, the training, validation, and test datasets encompassed a wider range of benign and malignant tumor types, enhancing generalizability. The relative contribution of each of these changes was not quantified, as no ablation study was performed.

HDI improves diagnostic performance by using active thermography, which applies a controlled thermal stimulus to assess dynamic physiological processes that differ between benign and malignant tissues and that cannot be captured by passive imaging. This approach is established in human oncology and, when combined with AI, enables the detection of subtle patterns beyond visual interpretation, supporting its relevance in veterinary oncology [16].

Clinically, the HDI system functions as a two-step triage tool. First, lesions are classified as benign or malignant with high sensitivity and NPV, providing confidence in ruling out malignancy. Second, tumor-specific classifiers add diagnostic refinement: the lipoma classifier offers high PPV and exceptional NPV for malignancy exclusion, while the MCT classifier identifies lesions with elevated malignant risk. This sequential strategy supports efficient clinical decision-making, possibly reducing unnecessary invasive procedures while ensuring that malignant tumors are appropriately prioritized. It is important to emphasize, however, that the HDI system is intended as a triage and screening aid, not as a substitute for cytological or histopathological diagnosis. Histopathology remains the diagnostic gold standard, as it alone can provide essential parameters such as mitotic index, depth of invasion, and tissue architecture, which are critical for accurate prognostic staging and treatment planning [24].

Several limitations should be acknowledged.

Statistical limitations. The relatively small size of the test set limited the statistical power of some subgroup analyses, and in some cases prevented predictive values from being estimated at all, as no false positives were observed for either tumor-specific classifier. Where values were therefore derived from the combined training and test sets, they include masses used during model development and should be interpreted with caution, as they may not fully reflect performance on independent data.

Reference standard and validation. This study relied on a mixed reference standard as most diagnoses were based on cytology, with a subset confirmed by histopathology. While cytology is clinically valuable, it may be subject to false-positive or false-negative results. Larger prospective studies incorporating histopathology as a uniform reference standard, and conducted across diverse clinical settings, are needed to further validate these findings. While cases were collected from diverse geographic sites, all data collection, analysis, and model development were conducted by a single research group. The classifier has therefore not yet been evaluated through independent validation by external investigators or institutions using separately governed data and workflows. This raises the possibility of undetected biases related to case mix, referral patterns, or operator technique across sites, and underscores the need for independent external validation.

Operational and technical limitations. Movement-related interruption during scanning may not be independent of lesion site or patient temperament, so some degree of selection bias cannot be excluded. Scans excluded due to motion artifact, technical failure, or incomplete clinical data were not fully characterized in this study, and we cannot exclude the possibility that excluded cases differed systematically from those retained for analysis. In addition, the HDI system’s performance is inherently operator-dependent: accurate results require proper clipping of the scan area, precise delineation of the tumor, buffer, and healthy-skin ROIs, and adequate patient restraint to minimize motion artifacts. Variability in technique between operators, and a potential learning curve for less experienced users, could affect data quality and downstream classifier performance, though this was not formally quantified here. Future work should include a multi-operator reproducibility analysis to characterize this variability and inform training requirements for clinical adoption.

The proprietary nature of the HDI classification algorithm further limits transparency into its internal decision-making, which may constrain independent interpretation, reproducibility, and error analysis by outside investigators.

This study evaluated diagnostic accuracy against a cytological or histopathological reference standard. It did not assess clinical utility, that is, whether use of the system alters diagnostic decisions, the number of invasive procedures performed, or patient outcomes. Statements regarding potential clinical application are therefore hypothesis-generating and require prospective evaluation in which clinicians act on the system’s output.

As the HDI platform continues to evolve and is trained on increasingly diverse datasets, further improvements in sensitivity, specificity, and tumor-type coverage are anticipated. Future work may expand tumor-specific classification, integrate multimodal data sources, and enable longitudinal monitoring of tumor progression. Addressing anatomical variability and validating models in larger heterogeneous populations will enhance robustness and generalizability, supporting broader clinical adoption of HDI in veterinary medicine.

## 5. Conclusions

Combining dynamic thermal imaging with artificial intelligence shows promise as a non-invasive triage aid for cutaneous and subcutaneous tumors in dogs. In this study, the primary classifier achieved high sensitivity and negative predictive value, suggesting strong potential as a screening tool to help rule out malignancy. The tumor-specific classifiers for lipomas and mast cell tumors added valuable subtype-specific information that may meaningfully facilitate early detection and guide clinical decision-making, including prioritization of cytological or histopathological follow-up. These encouraging findings mark a promising preliminary validation milestone and, while hypothesis-generating in nature, highlight the considerable potential of this approach to support veterinary workflows in general practice. Building on this strong foundation, future prospective studies, including evaluation of clinical utility and independent external validation, are well positioned to confirm and extend these results toward routine clinical application.

## Figures and Tables

**Table 1:** Diagnostic results of 1010 masses. Final diagnosis of all 1010 scanned masses, with the proportion of the total study population and the reference standard by which each diagnosis was established.

Diagnosis	n	%	Cytology	Histopathology ^a^
Benign lesions				
Lipoma	414	41.0	399	15
Benign adnexal/epidermal cyst/tumor	172	17.0	142	30
Sebaceous adenoma	45	4.5	37	8
Perineal adenoma	6	0.6	2	4
Hemangioma	5	0.5	2	3
Histiocytoma	4	0.4	1	3
Benign melanoma	3	0.3	2	1
Fibroma	2	0.2	0	2
Calcinosis circumscripta	1	0.1	0	1
Hyperplasia	1	0.1	0	1
Round cell tumor (benign)	1	0.1	0	1
Total benign	654	64.8	585	69
Inflammatory lesions	58	5.7	48	10
Malignant lesions				
Mast cell tumor	197	19.5	57	140
Soft tissue sarcoma	73	7.2	19	54
Carcinoma	9	0.9	1	8
Malignant melanoma	6	0.6	3	3
Hemangiosarcoma ^b^	5	0.5	0	5
Osteosarcoma	2	0.2	1	1
Sebaceous epithelioma	2	0.2	0	2
Histiocytic sarcoma	2	0.2	1	1
Cutaneous lymphoma	1	0.1	0	1
Plasma cell tumor	1	0.1	0	1
Total malignant	298	29.5	82	216
Total	1010	100	715	295

^a^ Forty-two of the 295 histopathologically confirmed masses also underwent cytology: mast cell tumor 20, soft tissue sarcoma 8, benign adnexal/epidermal cyst/tumor 6, lipoma 3, and one each of carcinoma, perineal adenoma, hemangioma, fibroma, and inflammatory process. Concordance between the two methods is reported in the Results. ^b^ Comprising three cutaneous and two subcutaneous hemangiosarcomas.

**Table 2:** Primary classifier results for benign masses in the training and test sets. Classification of the 654 benign masses by the primary classifier, shown separately for the training and test sets and by final diagnosis. The 58 masses of the inflammatory set are shown separately.

Tumor Class	Correct Benign Classification	False Malignant Classification	Total	Correctly Classified (%) ^a^
Training set				
Lipoma	287	54	341	84.2
Benign adnexal/epidermal cyst/tumor	61	79	140	43.6
Sebaceous adenoma	22	13	35	62.9
Perineal adenoma	0	6	6	0.0
Hemangioma	3	1	4	75.0
Histiocytoma	1	3	4	25.0
Benign melanoma	2	1	3	66.7
Calcinosis circumscripta	0	1	1	0.0
Fibroma	0	1	1	0.0
Hyperplasia	0	1	1	0.0
Round cell tumor (benign)	1	0	1	100.0
Total, training set	377	160	537	70.2
Test set				
Lipoma	62	11	73	84.9
Benign adnexal/epidermal cyst/tumor	13	19	32	40.6
Sebaceous adenoma	9	1	10	90.0
Fibroma	0	1	1	0.0
Hemangioma	0	1	1	0.0
Total, test set	84	33	117	71.8
Total, all benign masses	461	193	654	70.5
Inflammatory set ^b^	16	42	58	27.6

^a^ Percentages are the proportion of masses in each row correctly classified as benign by the primary classifier, that is, class-specific specificity. ^b^ The inflammatory set comprises 58 masses with a final diagnosis of inflammatory process, all benign, analyzed as a separate challenge group and excluded from the benign totals above.

**Table 3:** Primary classifier results for malignant masses in the training and test sets. Classification of the 298 malignant masses by the primary classifier, shown separately for the training and test sets and by final diagnosis.

Tumor Class	False Benign Classification	Correct Malignant Classification	Total	Correctly Classified (%) ^a^
Training set				
Mast cell tumor	9	135	144	93.8
Soft tissue sarcoma	5	49	54	90.7
Carcinoma	1	8	9	88.9
Hemangiosarcoma ^b^	2	3	5	60.0
Malignant melanoma	0	5	5	100.0
Histiocytic sarcoma	0	2	2	100.0
Sebaceous epithelioma	0	2	2	100.0
Cutaneous lymphoma	1	0	1	0.0
Osteosarcoma	0	1	1	100.0
Total, training set	18	205	223	91.9
Test set				
Mast cell tumor	3	50	53	94.3
Soft tissue sarcoma	2	17	19	89.5
Malignant melanoma	0	1	1	100.0
Osteosarcoma	1	0	1	0.0
Plasma cell tumor	0	1	1	100.0
Total, test set	6	69	75	92.0
Total, all malignant masses	24	274	298	91.9

^a^ Percentages are the proportion of masses in each row correctly classified as malignant by the primary classifier, that is, class-specific sensitivity. ^b^ Hemangiosarcoma comprises three cutaneous and two subcutaneous tumors.

## Data Availability

The original contributions presented in this study are included in the article/Supplementary Material. Further inquiries can be directed to the corresponding author.

## Supplementary Materials

The following supporting information can be downloaded at: https://www.mdpi.com/article/10.3390/vetsci13090932/s1; Table S1: Training hyperparameters for the focal thermal feature detection network; Table S2: Training hyperparameters for the optical mass depth detection network. Table S3: Confusion matrices and diagnostic performance for all classifiers.

## Abbreviations

AI	Artificial intelligence
DP	Decay point
FNA	Fine-needle aspiration
HDI	Heat-diffusion imaging
LED	Light-emitting diode
LWIR	Long-wave infrared
LR	Likelihood ratio
MCT	Mast cell tumor
NPV	Negative predictive value
PPV	Positive predictive value
ROC-AUC	Area under the receiver operating characteristic curve
ROI	Region of interest
