# Supplementary material for: Tumor-Specific Classification of Canine Cutaneous and Subcutaneous Tumors Using Heat-Diffusion Imaging and Artificial Intelligence
Source: Vet Sci. 2026 Sep 9;13(9):932. doi: 10.3390/vetsci13090932 (PMC13611836; doi:10.3390/vetsci13090932)
Supplement: Supplementary file 1 [file vetsci-13-00932-s001.zip › vetsci-4410810-supplementary.pdf]

Supplementary Table S1: Training hyperparameters for the focal thermal feature detection network.

**Hyperparameter**	**Value**
Optimizer	Adam
Learning rate	1×10^{-3}
Weight decay	1×10^{-5}
Initial learning-rate schedule	Reduce-on-plateau, factor 0.1, patience 5, cooldown 5, minimum 1×10^{-6}
Batch size	16
Maximum epochs	120
Early stopping	Patience 12 on held-out loss, best checkpoint restored
Augmentation	Random horizontal and vertical flips ($p = 0.5$), random rotation ±45°

Settings used to train the three-level convolutional encoder–decoder (U-Net) described in the Methods. Optimization minimized a per-pixel binary cross-entropy loss computed over annotated pixels only, with an additional penalty on predicted area to discourage over-segmentation. Data were partitioned at the scan level. Because early stopping was monitored on held-out loss with the best checkpoint restored, the maximum epoch count is an upper bound rather than the number of epochs actually trained.

Supplementary Table S2: Training hyperparameters for the optical mass depth detection network

Hyperparameter	Value
Backbone	DINOv2 ViT-S/14, shared backbone, fine-tuned end-to-end
Inputs	200 × 200 mass patch + 760 × 460 full optical frame, each resized to 224 × 224; features concatenated
Input resolution	224 × 224
Optimizer	AdamW
Learning rate	head 1×10^{-3}, backbone 1×10^{-5}
Learning-rate schedule	Cosine
Loss	Focal loss (γ = 1)
Class balancing	Square-root-frequency sampler and class weights
Batch size	16
Maximum epochs	50
Early stopping	Patience 12 on validation macro-F1
Decision rule	Argmax (threshold tuning tested; no improvement)
Seed	42

Supplementary Table S3. Confusion matrices and diagnostic performance for all classifiers.

Test set results are the primary analysis for each classifier; training-set and combined training and test results are shown for completeness. Confidence intervals (CI) are Wilson score, 95%. For both sub-classifiers, the actual masses are shown in three categories rather than two: the target tumor type, other masses of the same benign or malignant status, and masses of the opposite status.

A. Primary classifier: benign versus malignant

Actual	Predicted malignant	Predicted benign	Total
**Test set (n = 192)**			
Malignant	69	6	75
Benign	33	84	117
**Total**	**102**	**90**	**192**
**Training set (n = 760)**			
Malignant	205	18	223
Benign	160	377	537
**Total**	**365**	**395**	**760**
**Training and test (n = 952)**			
Malignant	274	24	298
Benign	193	461	654
**Total**	**467**	**485**	**952**

The inflammatory challenge group was not part of the training or test split and contains no malignant masses, so it is shown separately.

Actual	Predicted malignant	Predicted benign	Total
**Inflammatory group (n = 58)**			
**Benign (all masses)**	**42**	**16**	**58**

B. Lipoma sub-classifier

Masses assessed by the lipoma sub-classifier are those whose probability score exceeded the predefined threshold for benign classification.

Actual	Flagged lipoma	Not flagged	Total
**Test set (n = 55)**			

Actual	Flagged lipoma	Not flagged	Total
Lipoma (target)	38	6	44
Other benign	0	10	10
Malignant	0	1	1
**Total**	**38**	**17**	**55**
**Training set (n = 251)**			
Lipoma (target)	182	32	214
Other benign	2	33	35
Malignant	1	1	2
**Total**	**185**	**66**	**251**
**Training and test (n = 306)**			
Lipoma (target)	220	38	258
Other benign	2	43	45
Malignant	1	2	3
**Total**	**223**	**83**	**306**

C. MCT sub-classifier

Masses assessed by the MCT sub-classifier are those whose probability score exceeded the predefined threshold for malignant classification.

Actual	Flagged MCT	Not flagged	Total
**Test set (n = 81)**			
MCT (target)	14	30	44
Other malignant	2	14	16
Benign	0	21	21
**Total**	**16**	**65**	**81**
**Training set (n = 249)**			
MCT (target)	41	60	101
Other malignant	5	41	46
Benign	9	93	102
**Total**	**55**	**194**	**249**
**Training and test (n = 330)**			
MCT (target)	55	90	145

Actual	Flagged MCT	Not flagged	Total
Other malignant	7	55	62
Benign	9	114	123
**Total**	**71**	**259**	**330**

D. Prevalence-independent metrics

Classifier and set	Sensitivity % (95% CI)	Specificity % (95% CI)	LR+	LR−	ROC-AUC (95% CI)
Primary, test set	92.0 (83.6–96.3)	71.8 (63.0–79.2)	3.26	0.111	0.916 (0.867–0.955)
Primary, training set	91.9 (87.6–94.8)	70.2 (66.2–73.9)	3.09	0.115	0.884 (0.857–0.908)
Primary, training and test	91.9 (88.3–94.5)	70.5 (66.9–73.9)	3.12	0.114	0.890 (0.867–0.911)
Lipoma, test set	86.4 (73.3–93.6)	100.0 (74.1–100.0)	NE	0.136	0.969 (0.924–0.998)
Lipoma, training set	85.0 (79.7–89.2)	91.9 (78.7–97.2)	10.49	0.163	0.930 (0.862–0.980)
Lipoma, training and test	85.3 (80.4–89.1)	93.8 (83.2–97.9)	13.64	0.157	0.939 (0.885–0.980)
MCT, test set	31.8 (20.0–46.6)	94.6 (82.3–98.5)	5.89	0.721	0.643 (0.528–0.743)
MCT, training set	40.6 (31.5–50.3)	90.5 (84.7–94.3)	4.29	0.656	0.674 (0.600–0.747)
MCT, training and test	37.9 (30.4–46.0)	91.4 (86.4–94.6)	4.39	0.679	0.665 (0.599–0.723)

E. Prevalence-dependent metrics

Classifier and set	Prevalence-adjusted PPV %	Prevalence-adjusted NPV %	Observed PPV % (95% CI)	Observed NPV % (95% CI)	Accuracy % (95% CI)
Primary, test set	36.5	98.1	67.6 (58.1–75.9)	93.3 (86.2–96.9)	79.7 (73.4–84.8)
Primary, training set	35.3	98.0	56.2 (51.0–61.2)	95.4 (92.9–97.1)	76.6 (73.4–79.5)

Classifier and set	Prevalence-adjusted PPV %	Prevalence-adjusted NPV %	Observed PPV % (95% CI)	Observed NPV % (95% CI)	Accuracy % (95% CI)
Primary, training and test	35.5	98.0	58.7 (54.2–63.1)	95.1 (92.7–96.7)	77.2 (74.4–79.8)
Lipoma, test set	-	-	100.0 (90.8–100.0)	64.7 (41.3–82.7)	89.1 (78.2–94.9)
Lipoma, training set	-	-	98.4 (95.3–99.4)	51.5 (39.7–63.2)	86.1 (81.2–89.8)
Lipoma, training and test	-	-	98.7 (96.1–99.5)	54.2 (43.6–64.5)	86.6 (82.3–90.0)
MCT, test set	-	-	87.5 (64.0–96.5)	53.8 (41.9–65.4)	60.5 (49.6–70.4)
MCT, training set		--	74.5 (61.7–84.2)	69.1 (62.3–75.2)	70.3 (64.3–75.6)
MCT, training and test	-	-	77.5 (66.5–85.6)	65.3 (59.3–70.8)	67.9 (62.7–72.7)

LR+, positive likelihood ratio; LR−, negative likelihood ratio; MCT, mast cell tumor, NE, not estimable because no false positives were observed; ROC-AUC, area under the receiver operating characteristic curve. Prevalence-adjusted positive predictive value (PPV) and negative predictive value (NPV) are predictive values standardized to an assumed malignancy prevalence of 15%; these are not applicable to the sub-classifiers, whose target is a specific tumor type rather than malignancy. ROC-AUC confidence intervals were obtained by bootstrap resampling at dog level, 2,000 replicates.
